# Monthly Summary

**Published:** 1884-08

**Authors:** 


### Monthly Summary.

Cleanliness.—If the old adage be true that "Cleanliness is next to godliness," it is equally true that many dentists of the present day are very far away from godliness.

While it is not to be supposed that many gentlemen before me require a lecture upon refined manners and tidy habits, yet it is painfully true that many of our profession need just this very lecture upon a principle which is among the most important, if not *the most radical one of our calling*, and to which they would appear to be almost total strangers.

If the dentist does not possess sufficient means to furnish his office elegantly, he can certainly give it an air of cleanliness, and there is little excuse for allowing unsightly objects to confront and impress our patient at first sight with feelings of disgust. That this matter is not sufficiently considered by a large number of our brethren, proofs are very abundant.

There is no excuse for furniture covered with dust, carpets littered and unswept, or spittoons bespattered and unsightly with blood from a previous victim. There is no good reason why a dentist should not have a bountiful supply of clean napkins, towels and rubber dam. There is no good excuse for a dentist not thoroughly cleaning his forceps, his excavators, or his scalers and separating files. There is nothing to prevent a dentist from washing his hands and cleaning his nails before putting them in a patient's mouth. In fact, there is no reason why a dentist should not be as near perfection, in these preliminaries, as possible.

We are, to a great degree, *the educators* of our patients. If our habits are loose, our personal appearance untidy, and our operating room in a shabby and dirty state, we cannot complain if our patients are careless in their treatment of our place and its appurtenances ; if our hands and face and mouth are unclean, we dare not chide them for duty neglected and unperformed. Then, also, if a dentist be particular in keeping his office and its appurtenances neat and orderly, we feel he will be particular in having his operations thoroughly performed, and such an one will be most likely to gain confidence and esteem, even among entire strangers. In personal appearance, it is not necessary that a professional man should dress like a fop ; but clean linen and suitable attention to the toilet add much to personal charms.

Our patients are usually more observing than we suppose, and sometimes notice things that reflect unfavorably upon us ; hence, we need be on the alert, and see that our manners, our apparel and our office appointments are such as will bear scrutiny or defy criticism.

It was my privilege to visit the New York College of Dentistry, a short time since, and though I say it somewhat reluctantly, it is no less true, that it was about the dirtiest place and the most slovenly lot of persons I could conceive to be engaged in teaching the principles of dentistry, much less *its practice*. The demonstrators of operative dentistry, were not bad-looking individuals, but their personal appearance was decidedly against them, in my estimation. Greasy, shiny coats; linen spotted and soiled, hands grimy, and "nails with dirt enough under them in which to plant potatoes," as we used to to say when we were boys. Of course, the students, with such examples daily in their midst, were largely on the same plane with the demonstrators, and is it at all strange that, being with them from day to day in association, they should experience—perhaps unconsciously—an assimilation ?

Lastly, dentists should be clean and neat for *sanitary reasons*. Many diseases can be conveyed through the agency of instruments or appliances infected therewith. Instruments used in the mouth should always be washed thoroughly, and if any doubt exists in the mind of the operator concerning the presence of any contagious malady, the instruments should be disinfected by dipping them in a mild solution of carbolic acid and water.

Brethren, facts, like mules, are oftentimes stubborn things; but it becomes us, as professional men, as professional artists, and as professional teachers, to see to it that these important points be not slighted or overlooked. There is no need of mincing matters, and calling slovenliness *a manifestation of genius*, or dirt and disorder *the evidence of a good practice*. Carelessness and filthiness need no second invitation in a busy dentist's office—*we know* something about that. They are, therefore, no good evidence of professsional fitness but, as I have attempted to prove, are often the standard by which the proficiency by a dentist is judged. Let us, then, carefully guard this avenue of our reputation, and by precept and exambe diligent advocates and supporters of the habit of cleanliness. —*By Hyman Rosa, M. D. S., Kingston, N. Y.*

---

A VERY LARGE SALIVARY CALCULUS REMOVED FROM A PATIENT.—Salivary calculi are not of very rare occurance. A tumor under the tongue, on one side of frænum, a dilated duct from the sub-maxillary gland full of fluid, and called ranula, might contain calcareous deposits. But rarely have we the opportunity of reporting a calculus of such magnitude as the one which I extracted from the mouth of a patient two months since. I herewith append a report of the case:

Mr. W., aged 73, consulted me for a glaucomatous trouble. During the consultation, he desired to know whether his defect of vision could be aggravated by a disease of long standing in the mouth. In inspecting this cavity, I found a large swelling under the tongue, on the right side of the frænum. It was a prominent elevation on the floor of the buccal cavity, extending from the inner face of the chin toward the root of the tongue. Its nature was apparent at a glance, for at the anterior and upper part of this large, round, fleshy mass was an opening one-fourth of an inch in diameter, through which could be seen the exposed surface of a salivary calculus. A probe passed in through this opening entered into a large cyst, the thickened and distended duct of Wharton, which was filled with a solid calcareous mass. With a scissors I slit up the cyst wall, and removed from its bed a very large calculus of brownish white color, which weighed, when thoroughly dried, 159 grains. It was pear-shaped, the stem or contracted part having been moulded in the less dilated part of the salivary duct. The calculus was $1\frac{3}{8}$ inches long, $\frac{3}{4}$ of an inch broad at its largest end, and $\frac{3}{8}$ of an inch near its most contracted end.

A thread passed around the stone in its longest diameter measured $3\frac{8}{8}$ inches. The circumference at the thickest part was $2\frac{1}{4}$ inches, and $1\frac{1}{4}$ inches around its most contracted portion or stem. The surface of the stone was rough. It could be easily cut with a knife, the section showing a laminated structure. A drop of acid upon the surface caused carbonine acid to bubble up, showing that carbonate of lime entered into its composition. Chemical analysis showed that phosphate of lime was the chief constituent. The tumor had annoyed the patient for a long time. Its peculiar nature was not suspected till revealed by ulceration of the sac.—*Prof. Julian J. Chisholm, M. D., University of Maryland.*

---

CAST METAL PLATES.—For the lower jaw it is undoubtedly true that a "cast metal plate" of Watt's, Weston's, or Reese's metals is the best method for a majority of cases, especially where, as is so often the case, the absorption has been so great as to leave nothing but a thin shallow ridge of membrane, always more sensitive than the upper jaw, and which has, in a measure, to adapt itself to the plate.

By this process there is insured a better fit than we can be obtained by any other method, for it is cast to a non-shrinkage model of plaster and pumice, or sand, or whiting. (I prefer the former.)

It is so often, in this class of cases, that plates have to be filed to relieve irritation; with this work it can be done without marring its appearance.

I like the method of casting the plate to a pattern formed of sheet wax, the edges doubled all around, so that, when finished, it has the appearance of a band, as the rubber is finished flush to that edge. Then file and partially finish, try in the mouth, and cut away where necessary; spur the surface with a sharp graver, and attach the teeth with pink rubber. —*L. P. Haskell. Chicago, Illinois, in New England Journal of Dentistry.*

---

ARE THE ANTERIOR COLUMNS EXCITABLE ?—Van Deens first pronounced the view that the anterior colums of the spinal cord are not excitable. Fick proved by his experiments the contrary. But the majority of all investigators was in favor of Van Deens' view. Dr. Mendelssohn has recently tried to solve the disputed question in a different manner from his predecessors. In accordance with the physiology of the posterior columns, the fact is established that motion induced by their irritation is of reflex character. If, therefore, on irritation of the anterior columns, the motion in the muscles of the extremities produced by it should take place more rapidly, the response to the irritation be quicker, than that ensuing on irritating the posterior columns, we would have the proof that the anterior columns are directly excitable. Mendelssohn's experiments on frogs have really established this important fact. When he irritated the anterior columns of a frog, the motion ensuing in the extremities made its appearance far more quickly than was the case when he irritated the posterior columns.—*Deutsch. Med. Zeit.*, p. 409, 1884.

---

TOOTH BRUSHES: WHAT THEY SHOULD BE, AND WHAT THEY SHOULD NOT BE.—People usually select wrong tooth brushes. They are either too large or too hard. The usual aim is to "scrub" the teeth, but this is utterly wrong—the practice is pernicious and completely fails to cleanse the teeth, and remove food *debris*. People usually brush from side to side—*against the grain*—as it were. This custom tends to brush all the remains of food, etc., into the spaces between the teeth, where they rot and breed micro-organisms. The brush should be soft, should possess long vibrissœ and a long handle. it should be used *from* the gums towards the free end of the teeth, *i. e.*, downwards in the upper jaw, upwards in the lower. As to mouth-washes and dentifrices, they are mostly catchpenny impostures and most deleterious. Plain soap and plenty of water are best. Some people clean their teeth too often, some too seldom. *Clean* them before sleep and upon rising, *wash* them after meals, and if possible end the meal with a stiffish crust—is very sound advice which a German contemporary gives his readers.—*British Journal of Dental Science.*

---

MICROCOCCI CAUSING LOSS OF HAIR.—Without any detectable cause, and without the accompaniment of any other symptom, a male individual was attacked by alopecia, which the patient himself attributed to infection, contracted in a hair-dressing saloon. In the region of the right temporal bone was observed a circular place, 5 to 6 ctm. in diameter, perfectly bald. In its centre were noticed a few white hairs on the shiny skin, while the margin contained a zone covered by epidermis-scales. In this zone the hairs appeared broken and stunted in their growth. The least pull on a hair at once caused its falling out, the bulb always coming with it. By coloring, Dr. Sehlen, why reports this case in the *Centribl. f. d. Mediz. Wissensch.*, 15, 1884, was enabled to prove the presence of small, round cocci, which were less than a millimetre in size, and found in large quantities surrounding the hair in the cells of the epidermis, and sprinkled between the sheaths of the roots of the hair. The author has instituted a series of cultures not yet concluded. A mild solution of corrosive sublimate (1–3000) destroyed the cocci and established the re-growth of the hair.—*Med. and Surg. Reporter.*

---

MECHANICAL SKILL NECESSARY TO DENTISTS.—A physician may be very skillful without any mechanical skill, though a surgeon cannot be; but a dentist must have the element which the phrenologists, whose terms are better than their theories, call "constructiveness." Every dentist should have these six things: Good ability, good manners, a pleasant address, cleanliness, tact, and a good personal appearance. He is brought into close contact with his patrons, many of whom are ladies and children. He must touch them. He must encourage them; compose them when agitated, and help them to bear protracted, or, what is worse, sudden pain. —*Exchange.*

---

A DANGEROUS ADULTERATION OF IODOFORM.—Dr. Biel, of St. Petersburg, calls attention to a commercial adulteration of idoform with pieric acid, whtch cannot be detected by the tests of Pharmacopœia. This mixture is not only poisonous, but is explosive when rubbed up in a mortar. It can be detected by the citron-yellow color it yields to a watery filtrate. If a solution of cyanide of potassium be added to the filtrate, no reaction will follow if iodoform be pure; but if there be a trace of picric acid present, the sloution will in the course of ten minutes become brownish red (isopurpuric acid,) and in a short time deposits an insoluble precipitate of isopurpurate of potassium —*Deutsch American.*

---

THE CARE OF TEETH.—In a paper read before the Hygienic Society of Berlin, Herr Miller reported the results of his endeavors to determine the nature of the ferment occurring in the mouth, to which he is disposed to attribute the setting up of caries in the teeth. He stated that he had found the ferment to exist in the saliva, and that it appeared to result from the growth of two forms of lactic acid fungi which may prove to be reducible to a single species. In comparative tests as to the agents best suited to stop the development of the organism, and the consequent formation in the mouth of acid, to the action of which on the enamel of the teeth he considers the caries to be due, he found that a solution of corrosive sublimate, 1 in 500;000, checked the formation of acid, and a solution of 1 in 100,000 completely destroyed the organisms. He, therefore, thinks that a solution of corrosive sublimate, not strong enough to be dangerous, would be found a very effective mouth-wash. The spores of the organisms were found to remain capable of development after an hour's boiling in a solution of meat extract. The organisms were also destroyed by a solution of potassium permanganate, 1 in 1,000; solution of carbolic acid, 1 in 500; and salicylic acid, 1 in 125, while the development of acid was checked by solutions of half their strength.—*St. Louis Druggist.*

LOOSE TEETH ON PLATES.—Single teeth on partial rubber or celluloid plates sometimes loosen, especially when the tooth stands alone in a narrow place between the natural teeth. This can be prevented by using copper wire, No. 24, nearly taking one turn around one of the pins, then across to the other, taking one turn around that also, then twist the ends together, *not tightly* or the pins may be cracked out of the tooth. Be careful that the wire is imbedded in the rubber and covered when the plate is finished. The same plan can be sometimes used when the articulation makes a very thin plate necessary. By using No. 30 wire, add teeth with pins in the proper place, a rubber plate can be made as thin as a gold or silver one. A better plan is to hard solder a gold backing to the tooth and bend it to the proper angle to be firmly held by the rubber. A hole roughly punched through the backing will help the rubber to hold it. When suitable thickness can be given to the plate, the copper wire has the advantage of being inexpensive.—*Dental Review.*

